# A comparison of health-related factors between patients diagnosed with ME/CFS and patients with a related symptom picture but no ME/CFS diagnosis: a cross-sectional exploratory study

**DOI:** 10.1186/s12967-022-03769-x

**Published:** 2022-12-09

**Authors:** Gabriella Bernhoff, Eva Rasmussen-Barr, Lina Bunketorp Käll

**Affiliations:** 1 Department of Neurobiology, Care Sciences and Society, Division of Family Medicine and Primary Care, Karolinska Institute, Alfred Nobels allé 23 D2, Huddinge, 141 83, Stockholm, Sweden; 2 ME Centre, Bragée Clinics, Stockholm, Sweden; 3 Department of Neurobiology, Care Sciences and Society, Division of Physiotherapy, Karolinska Institute, Stockholm, Sweden; 4 Department of Health and Rehabilitation, Institute of Neuroscience and Physiology, Sahlgrenska Academy, University of Gothenburg, Göteborg, Sweden; 5 Centre for Advanced Reconstruction of Extremities, Sahlgrenska University Hospital/Mölndal, Mölndal, Sweden

**Keywords:** Central nervous system, Chronic pain, Public health, Biopsychosocial models, Patient reported outcome measures

## Abstract

**Background:**

In chronic fatigue syndrome/myalgic encephalomyelitis (ME/CFS), the capacity for activity and participation is strongly limited. The disease definition is very broad, and considering the lack of evidence for best treatment, it is important to understand what is ME/CFS-specific in the biopsychosocial perspective in comparison with similar syndromes. The objective was to study the difference between those diagnosed with ME/CFS and those with similar symptoms but no ME/CFS diagnosis for self-perceived level of physical activity, work ability, anxiety/depression, and health-related quality of life.

**Methods:**

This was a clinical cross-sectional study with data collected from mailed questionnaires. The following variables were compared between patients diagnosed with ME/CFS (n = 205) and those with similar symptoms but no diagnosis (n = 57); level of physical activity, Work ability index (WAI), Hospital anxiety and depression scale (HAD-A/HAD-D), and RAND-36 Physical functioning, Role limitations due to physical health problems, Role limitations due to personal or emotional problems, Social functioning, Energy/fatigue, Bodily pain, Emotional well-being, and General health perceptions. The Chi-squared test (nominal data), the Mann-Whitney U test, the Student’s t test and regression analysis were used to analyze the data.

**Results:**

The group diagnosed with ME/CFS had a more impaired physical and mental exertion ability as compared to the group that had similar symptoms but was not diagnosed with ME/CFS, shown by a RAND-36 lower index of physical role functioning, social functioning, energy, worse pain and poorer overall health (p ≤ 0.05). In contrast, no significant group differences emerged for weekly level of physical activity, work ability, anxiety/depression, and RAND-36 Emotional role limitation and well-being.

**Conclusion:**

Our results indicate that those with a diagnosis of ME/CFS are characterized by an impaired ability for physical or mental exertion, worse pain, and poorer overall health as compared to individuals with similar symptoms but for whom ME/CFS-diagnosis was not established. The results may be cautiously interpreted as support when focusing on patients’ self-care in terms of management of energy levels. The results must however be verified in future studies.

## Background

In recent years, chronic fatigue syndrome (CFS) or myalgic encephalomyelitis (ME), hereinafter referred to as ME/CFS, has been, and continues to be, a debated field [1–3]. In addition to possible pathomechanisms, special attention has been focused on the severely limited activity and restricted participation that follows from the in many cases severe disabilities [4, 5]. ME/CFS involves a persistent lack of energy or persistent pain, and often overwhelming fatigue from simple exertion [5]. Patients with ME/CFS often report that they cannot cope with chores at home, leisure activities or socializing with others [6, 7]. VanElzakker et al. [8] describe the underlying causes of the disease as essentially unknown, even if a substantial part of those who have developed ME/CFS did so after a severe viral or bacterial infection, where healing or recovery did not occur. Some evidence suggests links between central sensitization in ME/CFS and interactions between psychosocial parameters such as depression and level of physical activity [9, 10].

To formulate the diagnosis, the Canadian criteria based on seven symptoms are used: fatigue, exercise-induced deterioration, sleep disturbances, pain, neurological and cognitive symptoms, as well as autonomic, neuroendocrine and immunological symptoms, and the symptoms should have lasted for more than 6 months [4]. Even though early diagnosis is encouraged, the average time from symptoms to diagnosis is 3.6 years [11]. As a result of this delay, along with the lack of awareness and knowledge, there are reasons to believe that tens of millions in the world have undiagnosed ME/CFS [12]. According to The Swedish National Association for ME Patients (RME), there are about 10.000 people with ME/CFS in Sweden [13].

The definition and the understanding of ME/CFS is challenging. People living with ME/CFS report a feeling of being unsupported or dismissed by health professionals or employers who don’t take their symptoms seriously [14]. The pathophysiology of ME/CFS is in the form of neuropathies (dysautonomia and neuropathic pain), of a character or with manifestations that can be psychologized by both caregivers and the patients themselves, often influenced by health care contacts, in cases where the right knowledge is not available [15, 16].

To manage the complexities of patients, a more flexible biopsychosocial approach is recommended [17]. Considering the broad definition of ME/CFS, it would be of interest to define what characterizes those with established ME/CFS diagnosis regarding self-perceived health factors compared to those with a related complex symptom picture who do not have a diagnosis.

The aim of this study was therefore to investigate how patients referred to a specialist clinic for suspicion of ME/CFS, rated several health-related factors: perceived level of physical activity, work ability, anxiety/depression, and health-related quality of life. We also sought to investigate whether the factors differed between patients who met the criteria for ME/CFS diagnosis, and those where ME/CFS diagnosis was not established.

## Methods

The present work is an explorative clinical study in which participants (n = 277) were consecutively recruited among patients referred from primary care to a specialist ME/CFS clinic for evaluation of ME/CFS.

Patients whose referral were accepted by the specialist clinic were sent questionnaires by mail for a routine initial status (below). An invitation for study participation with research person information and a consent form was included. The mailing and receipt of questionnaires were handled by the receptionists at the clinic independently of the study.

The inclusion criteria were men and women (≥ 18 years) with a suspected ME/CFS diagnosis who were admitted for further investigation at the specialist clinic. No exclusion criteria were used, apart from those applied in the reviews of referrals, such as any clinical condition that would limit the ability to take part in the investigation (e.g. known drug abuse) or limited ability to speak and comprehend the Swedish language.

### ME/CFS diagnosis

The final diagnosis was established by the attending physician at the specialist clinic by mapping the patient’s current symptoms, using a structured clinical protocol, according to the Canadian diagnostic criteria for ME/CFS [4]. This included taking a patient history, conducting a physical examination, and evaluating lab tests as well as any differential diagnoses, to rule out other exclusionary diseases.

### Instruments

#### Physical activity

A physical activity questionnaire was used to specify the total time spent on activity during a normal week, for different aspects [18]. The first question asks about time spent on high intensity activity, and the second question deals with moderate intensity; time spent practicing everyday physical activity. Both have fixed answer options (time categories). The results from the two are merged, which generates an outcome of “activity minutes” (recommended level more or equal to 150 activity minutes per week). These questions have been evaluated for psychometric properties and were shown to be equivalent to other self-reported questions about physical activity [18].

#### Work ability index (WAI)

WAI is primarily used to identify signs of ill health in workers [19]. The instrument has 10 questions in 7 different areas [20, 21]. In the present study, only the first question, WAI 1, was used, for rating one’s own current work ability versus one’s best during one’s lifetime on a scale of 0–10. WAI 1 has been found to be a suitable proxy for the WAI and with acceptable validity [22–24]. With this item, the individual’s working capacity can be classified into poor (0–5), moderate (6–7), good (8–9) or excellent (10) working capacity [22].

#### Hospital anxiety and depression scale (HAD)

HAD has two subscales to obtain a measurement of anxiety symptoms and depressive symptoms (HADS-anxiety/worry, HADS-depressiveness/depression), extensively evaluated for psychometric properties [25, 26]. Each subscale has seven statements, in total 14 claims, all concerning the past week [27–29]. Answers are indicated on a four-point Likert scale (0–3 points). Scoring can thus amount to a maximum of 21 points per subscale. The patient’s sum score shows any of three “severity levels”: small, moderate, or high risk to have a depression or anxiety of clinical significance. A cut value of 8 has been shown to be the most optimal for detection while having sufficient specificity [25, 26]. Participants who have 11 points or more on a subscale are likely to have a condition of clinical significance [26]. Scores between 15 and 21 indicate a severe anxiety or depression. We used the individual’s subscale means to replace missing scores, provided no more than 2 items of a subscale were missing [30].

#### RAND-36

RAND-36 is used for forming a deeper understanding of how people living with a condition assess and adapt to their health situation [31, 32]. RAND-36 was formerly called SF-36 (Short Form − 36), which was developed in the United States by the RAND corporation within the” Medical Outcomes Study” (MOS), to reflect the World Health Organization’s (WHO) definition of health.

RAND-36 consists of 36 multiple choice questions spread over eight sub-scales and has adequate measurement properties [31, 33]. The eight subscales are Physical functioning, Role limitations due to physical health problems, Role limitations due to personal or emotional problems, Social functioning, Energy/fatigue, Bodily pain, Emotional well-being, and General health perceptions, and each generate an overall index of 0–100%, where 100% represents excellent health. (Emotional well-being and energy/fatigue have been used interchangeably with general mental health and vitality, respectively). Compared with how the SF-36 is scored, the scoring differs for two domains: Role limitations due to physical health problems and Role limitations due to emotional problems, with 5-point response choices in RAND-36 instead of option yes/no. Items that were left blank (missing data) were not taken into account when calculating the scale scores [34]. Hence, scale scores represent the average for all items in the scale that the respondent answered. We allowed 1 missing item per subscale [34].

### Statistical analysis

Data was summarized with descriptive statistics and presented with median (range), mean (standard deviation) frequency and percentages. Missing cases were reported as such for each variable and not included in the analyses.

The Mann-Whitney U test (range data) was used to test the null hypothesis for ordinal data, that there was no difference between the group diagnosed with ME/CFS compared to the group who did not receive this diagnosis [35]. Parametric tests were used for age and physical activity minutes. The Shapiro-Wilks test was used to test for normality [36]. Linear regression analysis was further used to control for or filter out any influence of the demographics sex and age on perceived health status [33]. For the variable physical role limitations, most patients had the value 0 and only 15 patients had higher values (25, 50 or 100). It was therefore converted to a dichotomous variable (0/1, 1 replacing values greater than 0) and logistic regression was applied. Regression analysis was done with the diagnosis status as independent variable, the demographics as covariates, and the health-related factors as the dependent variables.

No power calculation was performed as the study was considered to be explorative. The predefined research project sample size of 270 participants was however considered adequate. This was based on an estimated required minimum of approximately 100 participants on expected approximate averages and standard deviations of measurements for the included instruments and taking into account some loss of data.

Original p-values were adjusted for multiplicity using the Holm method [37]. Conclusions were based on the adjusted p-values. The significance level was set at p ≤ 0.05. All data analyses were done in R version 4.1.1 [38].

## Results

A total of 277 patients were eligible for this study. For fifteen patients, the investigation was prematurely suspended due to changed priorities in the patients’ situations (e.g. to move elsewhere). The study group thus comprised 262 patients, of whom 205 (78%) were diagnosed with ME /CFS at the specialist clinic. The amount of missing data was < 10% for all variables (mixed item/unit non-response and lost files), as follows: 8 cases for the physical activity questions, 7 cases for the WAI, 6/6 cases for the sections on anxiety/depression. Regarding the RAND-36, it was not completed by 17 patients (Table 1).

**Fig. 1:**
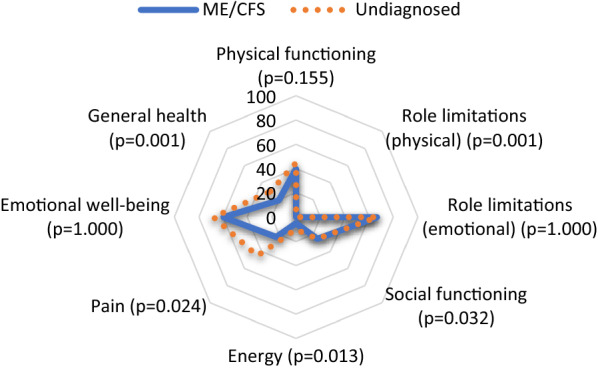
Self-perceived level out of the best possible level (100%), for the factors measured with RAND-36, with between-group differences for physical role and social functioning, energy, pain, and general health. Data shown are group median percentage

Characteristics of the diagnosed and undiagnosed group are presented in Table 1, together with median values of the self-reported level of physical activity, work ability, anxiety and depression, and health-related quality of life. Results showed significantly worse ratings in the group that was diagnosed with ME/CFS for the following RAND-36 outcome measures: decreased physical role functioning and vitality (energy/fatigue); decreased social functioning; worse pain; and lower general health perceptions (p ≤ 0.05) (Fig. 1). Apart from the significant relations to the ME/CFS diagnosis status, the reported health factors were generally not associated to demographics, with some exceptions: RAND physical functioning and bodily pain was associated with sex and general health was associated with age.

No between group differences were found for physical activity level, work ability, anxiety and depression, and for the RAND-36 role limitations due to personal or emotional problems, and emotional well-being.

**Table 1:** Characteristics of the study participants (n = 262) and group differences between those with and without a ME/CFS diagnosis for the measured variables

	Diagnosed ME/CFS (n = 205)	Undiagnosed (n = 57)	Missing cases. n (diagnosed /undiagnosed)	p	Adjusted p^a^	Adjusted P^b^ (Holm)
Gender (men/women), n (%)	25/180 (12/88)	18/39 (32/68)	–	0.001	–	0.013
Age (years), Mean (SD)	45 (11)	49 (14)	–	0.025	–	0.177
Physical activity Activity minutes weekly Mean (SD)	109 (107)	150 (141)	8 (6/2)	0.046	0.031	0.276
Work ability WAI score (0–10) *#	1.0 (0.0–3.0) 1.6 (1.9)	2.0 (0.0–4.5) 2.6 (2.5)	7 (5/2)	0.012	0.007	0.107
Anxiety HAD-Anxiety (0–21) *#	7 (3–11) 7.5 (4.8)	7 (4–9) 6.9 (4.3)	6 (6/0)	0.388	0.593	1.000
Depression HAD-Depression (0–21) *#	9 (6–13) 9.3 (4.7)	8 (6–11) 8.7 (4.5)	6 (6/0)	0.314	0.211	1.000
RAND-36						
Physical functioning (0-100) *#	40 (25–55) 40.3 (21.1)	45 (35–62) 48.9 (22.0)	15 (10/5)	0.019	0.029	0.155
Role limitations due to physical health problems (0/> 0) n (%)	189 (97)/6 (3)	42 (82)/9 (18)	16 (10/6)	< 0.001	0.001	0.001
Role limitations due to personal or emotional problems (0-100) *#	67 (0–100) 56 (45.4)	67 (0–100) 57 (44.3)	17 (11/6)	0.776	0.775	1.000
Social functioning (0-100) *#	25 (0–25) 21.3 (18.5)	25 (13–44) 32.0 (23.9)	8 (6/2)	0.003	0.001	0.032
Energy/fatigue (0-100) *#	5 (0–15) 10.3 (12.0)	10 (5–27.5) 19.4 (20.8)	6 (4/2)	0.001	< 0.001	0.013
Bodily pain (0-100) *#	23 (10–45) 30.7 (23.5)	45 (23–55) 41.4 (25.8)	7 (5/2)	0.002	0.011	0.024
Emotional well-being (0-100) *#	60 (44–76) 57.9 (21.5)	68 (44–76) 59.8 (22.4)	6 (4/2)	0.399	0.534	1.000
General health perceptions (0-100) *#	20 (15–30) 22.9 (13.6)	30 (20–43.8) 32.54 (17.5)	9 (6/3)	< 0.001	< 0.001	0.001

*HAD* Hospital anxiety and depression scale. *ME/CFS * myalgic encephalomyelitis/chronic fatigue syndrome. *Q1–Q3 * First and third interquartile range, *SD*  Standard deviation, *WAI* Work ability index

*Mdn (Q1–Q3). # Mean (SD)

^a^All p-values were adjusted for sex and age

^b^p-values were adjusted for multiple testing using the Holm method

## Discussion

Measuring of the various health-related factors showed that the group diagnosed with ME/CFS distinguished itself compared to the group of individuals for whom an ME/CFS-diagnosis was not established by an impaired ability for physical or mental effort (measurements of physical role and social functioning, and energy), worse pain and worse general health. No significant between-group differences were however found regarding assessed activity minutes per week, work ability, anxiety/depression, emotional role limitation and well-being.

The findings of impaired physical ability being characteristic in ME/CFS are, as could be expected, in accordance with previous studies [39]. A few previous studies have used the RAND-36 and indicated a slightly better self-perceived health among participants with ME/CFS than in the present study [40–42]. We reflect that this may in part be an effect of the broad diagnostic criteria for ME/CFS; one study had a group who met the Oxford criteria [40], and the other, both the Fukuda and Canada criteria [41]. The results of the present study support the role of the RAND-36 subscales Physical role limitation and Social functioning in the diagnostics of ME/CFS, as it showed outcomes that were consistent with the divide in the assessment where the diagnosis was or was not given. This was also acknowledged in a previous American study with a comparable population (70% women, average age 50 years) who met both the Fukuda and Canada criteria [43]. The authors recommended these sub-scales, as well as the sub-scale Physical functioning, for identifying the cardinal ME/CFS symptoms of impaired physical and mental ability [43].

The scoring of emotional or psychological ill-health (measurements of anxiety/depression, and emotional role limitations and well-being) reported in the present study was at the lower end (equals better health status) of reports in previous studies of the same population, which showed mean HAD-anxiety score 10.0 and mean HAD-depression 8.9 [44], while a study with patients with fibromyalgia [45] showed median HAD-anxiety 10.6 och HAD-depression 9.9. Surprisingly, no differences were found in our study between the diagnosed and the undiagnosed group regarding anxiety/depression, emotional well-being and emotional role limitation, so presumably none of these factors should have had an impact on the particularly low perceived energy level seen within the ME/CFS Group.

Central sensitization has been considered to be a common denominator or a main feature of chronic fatigue and associated conditions, such as whiplash associated disorders, but with different degree of severity, with ME/CFS placed at the far end of the scale and thus characterized by pronounced central sensitization [46]. Post-exertional malaise (PEM) is a manifestation of central sensitization [46]. Wormgoor et al. discuss the related diagnostic concepts and propose to define “ME” with post-exertional malaise (PEM) being included as a cardinal symptom, “CFS” with PEM occurring to varying degrees, and “chronic fatigue” with not including PEM [47]. The present study suggests a support for such a division of diagnoses.

Considering what would be the underlying mechanisms of the eroded physical functioning and energy in ME/CFS raises questions about this group’s pattern of activities of daily living and recovery. We hypothesize that a difficult combination of two precipitating factors could act in the development and maintenance of the disease in many cases. Firstly, an imbalance of activity–recovery being present in those diagnosed with ME/CFS leading to chronic physiological stress and a secondary dysautonomia as suggested by Martínez-Martínez [48]. There may be difficulties in completing activities, where the person has tried to perform activities based on old habits and routines such as before the illness, and difficulty managing energy levels [6]. One reason for this could be that the phenotype with overly elastic connective tissue is overrepresented in ME/CFS [49] and often accompanied by decreased interoceptive ability (ability to read, interpret and adapt to bodily signals) [50–54]. Secondly, a greater exposure from persistent negative psychosocial stress especially in ME/CFS, where the often-unexplained symptoms of ME/CFS may cause a notion of one’s well-being as unpredictable and out of control, leading to feelings of, among others, insecurity and a dependency on external circumstances [55]. A telling example is of recommendations from health care professionals to engage in general physical exercise, without the required adaptation of the training dose, where ME/CFS was not seriously considered as a diagnosis. This usually leads to exacerbation [56, 57]. Thus, the trust in one’s own abilities is undermined, with far-reaching, likely incalculable consequences.

According to clinical experience, this unfortunate combination is not seldom a challenge for patients with ME/CFS that can act detrimental to well-being. Not least does this apply to the illness’ traits pain and fatigue, since the body’s response to long-term stress includes the hypothalamic-pituitary-adrenal (HPA) axis being activated to affect nociception and with central sensitivity syndromes as a result [58, 59].

It is recommended for patients with ME/CFS and caregivers to work together to explore best practices [12, 17]. A biopsychosocial or holistic approach is part of the main recommendations in guidelines for ME/CFS [39]. The praxis, however, of a biopsychosocial management in ME/CFS is at the horizon, partly because of well-functioning multiprofessional teams not always being accessible in the primary health care system to which patients with ME/CFS are generally affiliated [60]. Also, many patient representatives have historically voiced a demand for development of the biomedical type of treatments in particular [61]. Finally, the mode of treatment of pacing or activity management needs to be further researched.

We used several PROMs to measure the health-related factors. The PROMs are commonly used in primary and secondary care in Sweden for various types of pain disorders and the outcomes are registered and followed up nationally in a quality registry of pain rehabilitation [62]. PROMs used in the assessment of ME/CFS are suggested to have some need for development, mainly to be made more relevant for the patient group [63]. Of the PROMs used in this study, only the HAD has been evaluated for its applicability among adults with suspected ME/CFS and was found suitable for this group of patients [64, 65].

A strength with this study is that it sheds light on the disease entity of ME/CFS. Self-perceived health has been extensively studied for various diseases, although to a relatively small extent for ME/CFS. Moreover, as far as we the authors are aware, this is the first study to compare diagnosed cases with ME/CFS and those with similar symptoms but no ME/CFS diagnosis from the same population. The results should, however, be interpreted in the light of the study’s limitations, first and foremost the cross-sectional nature of the study, and in which data was collected through patient-reported questionnaires. The group diagnosed with ME/CFS had a higher percentage of women than the undiagnosed group. If the groups would have been matched with respect to gender, the differences between the groups might have appeared otherwise since women, as a rule, have pain to a greater extent than men. Another limitation is that the diagnosis of ME/CFS was established from the recommended Canada criteria but also from subjective assessments depending on the experience of assessors that should be taken into consideration.

## Conclusion

Our results indicate that those with a diagnosis of ME/CFS are characterized by an impaired ability for physical or mental exertion, worse pain, and poorer overall health as compared to individuals with similar symptoms but for whom ME/CFS-diagnosis was not established. The results may be cautiously interpreted as support when focusing on patients’ self-care in terms of management of energy levels. The results must however be verified in future studies.

## Data Availability

The datasets used and analysed during the current study are available from the corresponding author on reasonable request.

## Abbreviations

CFS: Chronic fatigue syndrome
HAD: Hospital anxiety and depression scale
Mdn: Median
ME: Myalgic encephalomyelitis
Q1–Q3: First and third interquartile range
SD: Standard deviation
WAI: Work ability index
